# Recrudescence of Focal Stroke Symptoms during Pain Management with Hydromorphone

**DOI:** 10.3389/fneur.2016.00050

**Published:** 2016-03-31

**Authors:** Joshua D. Bernstock, Craig S. Budinich, Leonardo G. Cohen, Oluwole O. Awosika

**Affiliations:** ^1^Stroke Section, National Institute of Neurological Disorders and Stroke (NINDS), National Institutes of Health (NIH), Bethesda, MD, USA; ^2^Department of Anesthesiology, Dwight D. Eisenhower Army Medical Center, Fort Gordon, GA, USA; ^3^Human Cortical Physiology and Stroke Neurorehabilitation Section, National Institute of Neurological Disorders and Stroke (NINDS), National Institutes of Health (NIH), Bethesda, MD, USA

**Keywords:** stroke, pain management, opioids, plasticity, recrudescence of symptoms

## Abstract

Patients who have recovered from a prior stroke may experience a reemergence of their original stroke syndrome secondary to metabolic derangements, sedation, infection, and/or fatigue. Critically, the molecular/cellular mechanisms mediating symptom recurrence after exposure to analgesic agents remain unknown. Accordingly, herein, we report a unique case of a patient with hydromorphone-induced recrudescence 30 years after her initial stroke event(s) and in so doing propose a putative mechanism related to post-infarction functional neuroplasticity.

## Introduction

**Figure 1:**
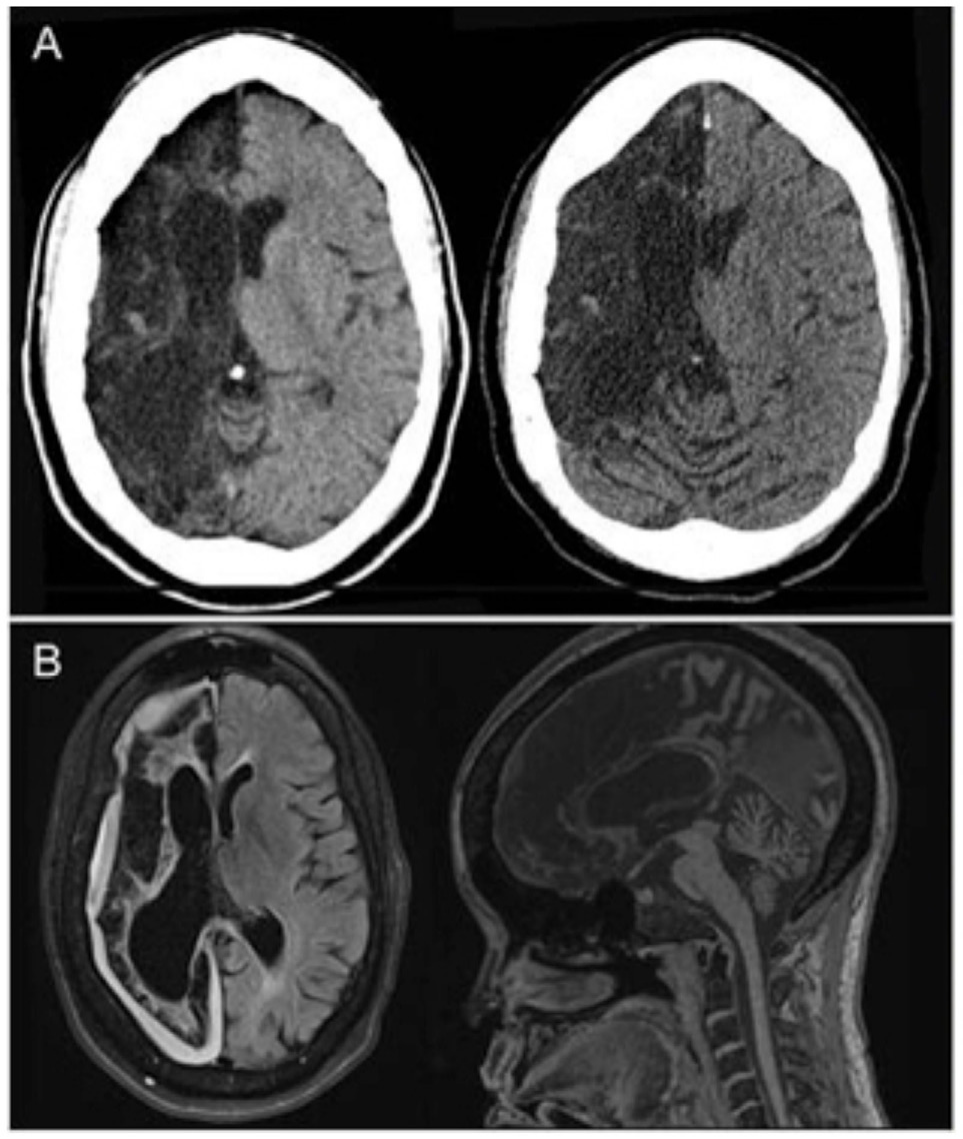
**(A)** Non-contrast head CT (*transverse views*) shows chronic right anterior cerebral artery (ACA) and middle cerebral artery (MCA) territory encephalomalacia with sparing of a small portion of the medial right frontal lobe, without evidence of bleed or new territory of acute infarction. **(B)** Both transverse and midsagittal MRI views obtained during an identical clinical episode from 3 years prior were likewise unremarkable for acute pathology.

A 44-year-old right-handed woman of Jamaican descent with homozygous sickle-cell anemia complicated by multiple right hemispheric strokes (occurring between the years 1983 and 1986) presented with 1 week of 9/10 refractory pain. She was diagnosed with a vaso-occlusive pain crisis and admitted to the telemetry floor for pain management and hydration. Her treatment regimen included a basal hourly rate of 0.5 mg intravenous (IV) hydromorphone (Dilaudid^®^) and patient-controlled analgesia (PCA) at 0.5 mg IV per 15 min. Within hours of admission, she was found unresponsiveness and bradypnic. Rapid response was initiated, and hydromorphone was immediately discontinued. Blood pressure and pulse remained stable near baseline 130 s/70 s mmHg and 70 s, respectively. Two doses of naloxone (Narcan^®^), an opioid receptor antagonist, were administered, with gradual improvement of her mental status and respiratory rate. STAT non-contrast head computed tomography (CT) was negative for acute intracranial pathology (Figure 1A). No additional interventions were made, and she was subsequently transferred to the intensive care unit (ICU) for overnight monitoring, where she remained hemodynamically and clinically stable. By morning, her somnolence had resolved and respiratory rate was stable at 16–17 breaths per minute; however, she endorsed the inability to move the left side of her body. Although fully alert and oriented, she was highly inattentive and perseverative. Cranial nerve examination was notable for sluggishly reactive pupils, left homonymous hemianopsia, and a dense left upper motor neuron (LUMN) facial droop. Motor exam (summarized in Table 1) demonstrated increased tone and spasticity on the left, with minimal voluntary movement in the left upper and lower extremities. Moreover, she displayed ipsilateral extinction to double simultaneous stimulation (DSS) and decreased sensation to light touch and pinprick on the left side of the body. Reflexes were brisk in the left upper and lower extremities, with a corresponding Babinski reflex. STAT labs were unremarkable for acute toxic-metabolic derangements. Moreover, there were no witnessed seizure-like events and/or related symptomatology to suggest a postictal paresis. Detailed review of her past medical records exposed an identical situation, which occurred approximately 3 years earlier, in the setting of a hydromorphone infusion. A full work-up at that time, including magnetic resonance imaging (MRI) (Figure 1B) and an electroencephalogram (EEG), were unrevealing, with symptoms having resolved approximately 24 h after discontinuing hydromorphone. Likewise, the presenting focal deficits witnessed resolved approximately 24 h after cessation of the offending agent (1, 2) (Table 1).

**Table 1:** **Focused cranial nerve and motor examination as a function of time after discontinuation of hydromorphone demonstrates transient worsening of the neurological exam with progressive return to baseline at 30 h**.

Examination	Time after hydromorphone intoxication							
	Baseline		0 h		10 h		30 h	
Pupils	3 → 2 mm briskly reactive bilaterally		3 → 2 mm sluggishly reactive bilaterally		3 → 2 mm sluggishly reactive bilaterally		3 → 2 mm briskly reactive bilaterally	
Face	Symmetric		Severe left UMN facial droop		Mild left UMN facial droop		Symmetric	

**Extremities**	**Right**	**Left**	**Right**	**Left**	**Right**	**Left**	**Right**	**Left**

Deltoids (C5)	5	2+	5	1	5	1	5	2+
Biceps (C6)	5	2+	5	0	5	1	5	2+
Triceps (C7)	5	2+	5	0	5	1	5	2+
Grip (C8/T1)	5	2+	5	0	5	1	5	2+
Iliopsoas (L2)	5	4+	5	0	5	2	5	4+
Quadriceps (L3)	5	4+	5	2	5	2	5	4+
Hamstrings (L4-S1)	5	4+	5	1	5	2	5	4+
Tibialis anterior (L4)	5	4	5	0	5	2	5	4
Extensor hallucis longus (L5)	5	4	5	0	5	2	5	4

## Background and Discussion

Herein, we present the case of a woman who developed severe acute focal neurologic deficits in the setting of hydromorphone toxicity. Interestingly, the deficits gradually resolved upon cessation of the drug and the allowance of time for kinematic clearance, recapitulating an earlier documented clinical event under a similar clinical context. Presently, the mechanisms behind hydromorphone-induced stroke recrudescence remain unknown. Early literature suggested a detrimental role of opioids in ischemic stroke (3–5), related to its ostensible hemodynamic and metabolic effects (6, 7). However, recent literature supports an alternative mechanism involving opioid receptor-mediated perturbation of neurotransmitter-specific neural networks critical to neuronal plasticity and recovery (8, 9). Specifically, following neurologic injury, a period of spontaneous recovery occurs, leading to the formation of new compensatory networks in perilesional, second-order, and homologous contralesional cortices (10, 11). Such recovery pathways are mediated/stabilized *via* excitatory glutamate-dependent *N*-methyl-d-aspartate (NMDA) and α-amino-3-hydroxy-5-methyl-4-isoxazole propionic acid (AMPA) receptors, leading to the downstream facilitation of both neurogenesis and synaptogenesis (12). Interestingly, work has emerged to suggest that opioid-dependent Mu Receptors (MOP-R) may interfere with and in so doing destabilize such compensatory neuroplasticity *via* direct inhibition of presynaptic glutamate, substance P, and/or neurokinin release, while further disrupting postsynaptic inhibition of second-order complexes essential for the maintenance of relatively nascent neural networks (13, 14).

Such MOP-R-meditated interference parallels our clinical observations being that the focal and highly stereotyped findings described within our patient cannot be explained merely by the effects of general sedation alone. Therefore, our interpretation of the reported clinical findings is that excessive opioid exposure led to the functional suppression of reorganized compensatory cerebral networks. Of note, a similar reemergence of stroke deficits has been well described following benzodiazepine (GABAA receptor agonist) administration (15–17). Furthermore, Thal et al. described a similar phenomenon in a cohort of patients with previously resolved neurologic deficits, who went on to develop the transient recurrence of prior deficits after administration of fentanyl (a synthetic opioid analgesic) and/or midazolam within months of the initial onset of their neurologic deficits (18).

It is prudent to note that an EEG was not obtained during this episode of recrudescence. As such, we are unable to definitively rule out the possibility of postictal paresis. However, the absence of a documented seizure history coupled with an unremarkable EEG during a similar clinical episode supports our assessment.

## Conclusion

This case reported herein is unique in that our patient’s prior motor deficits reoccurred more than 30 years after her initial stroke events, thereby implying that the aforementioned compensatory neural networks may in fact remain susceptible well beyond the period of functional recovery. While preliminary, this case further supports the notion that opioid analgesics may interfere with NMDA/AMPA-receptor transmitter-specific neural networks, resulting in the recrudescence of recovered deficits, and should therefore be administered cautiously in patients with known central nervous system lesions.

## Ethics Statement

Written informed consent was obtained on admission, which guarantees the ethical approval of the protocol the patient was admitted under. The study was approved by National Institutes of Health – Clinical Center.

## Author Contributions

JB, CB, LC, and OA wrote/revised the case report.

## Conflict of Interest Statement

The authors declared no potential conflicts of interest with respect to the research, authorship, and/or publication of this article.
